# Diversity of Meiofauna from the 9°50′N East Pacific Rise across a Gradient of Hydrothermal Fluid Emissions

**DOI:** 10.1371/journal.pone.0012321

**Published:** 2010-08-10

**Authors:** Sabine Gollner, Barbara Riemer, Pedro Martínez Arbizu, Nadine Le Bris, Monika Bright

**Affiliations:** 1 Department of Marine Biology, University of Vienna, Vienna, Austria; 2 Deutsches Zentrum für Marine Biodiversitätsforschung, Forschungsinstitut Senckenberg, Wilhelmshaven, Germany; 3 Department Environnement Profond, Institut français de recherche pour l'exploitation de la mer, Brest, France; Northern Fisheries Centre, Australia

## Abstract

**Background:**

We studied the meiofauna community at deep-sea hydrothermal vents along a gradient of vent fluid emissions in the axial summit trought (AST) of the East Pacific Rise 9°50′N region. The gradient ranged from extreme high temperatures, high sulfide concentrations, and low pH at sulfide chimneys to ambient deep-sea water conditions on bare basalt. We explore meiofauna diversity and abundance, and discuss its possible underlying ecological and evolutionary processes.

**Methodology/Principal Findings:**

After sampling in five physico-chemically different habitats, the meiofauna was sorted, counted and classified. Abundances were low at all sites. A total of 52 species were identified at vent habitats. The vent community was dominated by hard substrate generalists that also lived on bare basalt at ambient deep-sea temperature in the axial summit trough (AST generalists). Some vent species were restricted to a specific vent habitat (vent specialists), but others occurred over a wide range of physico-chemical conditions (vent generalists). Additionally, 35 species were only found on cold bare basalt (basalt specialists). At vent sites, species richness and diversity clearly increased with decreasing influence of vent fluid emissions from extreme flow sulfide chimney (no fauna), high flow pompei worm (S: 4–7, H'_loge_: 0.11–0.45), vigorous flow tubeworm (S: 8–23; H'_loge_: 0.44–2.00) to low flow mussel habitats (S: 28–31; H'_loge_: 2.34–2.60).

**Conclusions/Significance:**

Our data suggest that with increasing temperature and toxic hydrogen sulfide concentrations and increasing amplitude of variation of these factors, fewer species are able to cope with these extreme conditions. This results in less diverse communities in more extreme habitats. The finding of many species being present at sites with and without vent fluid emissions points to a non endemic deep-sea hydrothermal vent meiofaunal community. This is in contrast to a mostly endemic macrofauna but similar to what is known for meiofauna from shallow-water vents.

## Introduction

Marine communities and ecosystem processes are affected by environmental changes, such as global warming, ocean hypoxia, and ocean acidification [1]. Stress, defined as reduction of an organism's potential growth, is one of the key components ruling diversity [2], [3]. According to ecological theory, across an environmental stress gradient highest diversity is expected at intermediate stress levels. At lower stress levels dominant species are encouraged to consume all resources, whilst at higher levels of stress only colonizing species survive [4]–[6].

Deep-sea hydrothermal vents are physically highly disturbed and stressful marine environments [7]. Dramatic and unpredictable catastrophic volcanic eruptions, tectonic disturbances, rapid changes in vent fluid composition, and the dynamics of waxing and waning vent fluids characterize this ecosystem [8], [9]. Highly variable physico-chemical conditions such as high temperature and pH gradients, the enrichment in toxic chemicals and the intermittent availability of oxygen impose physiological stress to animals living at such extreme conditions. Stress results in reduced rates of biochemical reactions when conditions are outside the optimal range of tolerance [5], [10].

Hydrothermal vents are relatively small and patchy habitats within the axial summit troughs (AST) of the mid-ocean ridges - large, continuous and scarcely populated bare basalt surfaces. Vents represent islands where chemosynthetic primary production locally supports high macrofauna abundances [7]. Primary production is carried out by chemolithoautotrophic bacteria, using the energy provided by the mixing of the reducing hydrothermal fluid emissions and oxygenated seawater to fix inorganic carbon [11]. As part of the free-living microbial community they are the foundation of the food web at vents. As symbiotic partners, they occur in a variety of associations with animals such as bathymodiolin mussels, vestimentiferan tubeworms, or alvinellid polychaetes [12]. These symbioses often function as foundation species in creating and structuring the habitat, modifying their environment by changing the physical and chemical properties, concentrating food sources, and thus providing space for associated fauna [7], [10].

Worldwide, over 500 animal species have been described from hydrothermal vents and over 90% are considered endemic to this ecosystem [13]–[15]. In hydrothermal vent research the word “endemic” is often used for species that are restricted to the vent environment and not for species within a certain geographical region, according to its original definition [16]. The vent macrofauna communities are generally characterized by low diversity and low species richness but high abundances [17]–[20].

One of the best-known mid-ocean ridge regions is located at 9°50′N, 104°17′W on the East Pacific Rise (EPR) with a fast spreading rate of 55 mm yr^−1^ [21]. The ridge crest is broad and shallow and lies at a depth of about 2500 m. An axial summit trough (AST) with associated lava channels is ∼50 m wide and ∼20 m deep [9]. Vent communities at the 9°50′N region are known to be frequent and diverse [22], [23]. The occurrence of characteristic foundation species and associated communities shows a striking spatial distribution pattern along a thermal and chemical gradient of hydrothermal fluid emissions. Several habitat types associated with different styles of venting can be distinguished: high temperature flow (>50°C) with alvinellid polychaetes colonizing sulfide chimneys (e.g. pompei worm *Alvinella pompejana* and *A. caudata*), vigorous, but moderate temperature flow (<30°C) with vestimentiferans (e.g. tubeworm *Riftia pachyptila*) growing on basalt, low temperature flow (<15°C) with bivalves (e.g. bathymodiolin mussel *Bathymodiolus thermophilus*) on basalt, and very low or no detectable vent flow with suspension feeders (serpulids, barnacles, anemones) [18], [24]–[27]. In addition, there are habitats with no visible fauna such as the high temperature (up to 400°C) areas of bare sulfide chimneys and bare basalt habitats with no direct influence of hydrothermal fluid emissions and ambient deep-sea temperature [7].

Vent fluid temperature is not the only parameter discriminating the habitats of foundation species. In the pompei worm habitat, pH is lower (∼ down to pH 4) and toxic sulfide concentration (∼ up to 1500 µM sulfide) is much higher for a given temperature than at any other vent habitat [26], [28]. In contrast, the temperature-sulfide relation is more consistent within vigorous and low flow habitats where vestimentiferan tubeworms and bathymodiolin mussels are found. There, sulfide concentrations range from ∼100 to 300 µM and pH ranges from ∼4 to 7 [25]. In addition, variations of oxygen concentrations and other oxidized compounds create temporarily anoxic, hypoxic, and oxic conditions at vent habitats [29], [30]. At bare basalt where venting is absent, environmental conditions are similar to those of the surrounding deep-sea water, i.e. no sulfide is detectable, oxygen, pH and temperature are close to ambient [31].

Not only high temperatures, high sulfide concentrations, and low pH, but also the rapid variations of these parameters are characteristic for the vent ecosystem. In the pompei worm habitat temperatures from ambient (∼2°C) up to 100°C are reported. Variations of 10 to 20°C over a few seconds/minutes and temperature spikes of up to 40°C are frequently observed [26], [32]. In the more moderate tubeworm habitat overall temperature ranges can be up to 30°C, and variations up to 15°C within seconds are common [25], [33]. In the low flow mussel habitat quick temperature variations of ∼5°C are reported [33]. Also pH and sulfide concentrations are changing on a second and/or minute timescale. In addition, spatial variations of hydrothermal fluid emmisions are found on a centimeter scale [25].

The zonation of foundation species along this physico-chemical gradient was initially attributed to physiological responses to stress and nutrient requirements [8], [23], [31]. Since then, biological factors such as competition and predation, facilitation and inhibition were found to mediate the limits of distribution. It has been concluded that correlations with abiotic gradients provide insufficient evidence for inferring causation of zonation along environmental gradients [34]–[36].

Our knowledge on diversity of epizooic macrofauna (animals larger than >1 mm) associated with foundation species of the 9°50′N EPR is limited to tubeworm and mussel habitats. Two chemically different sites colonized by tubeworms exhibited similar macrofaunal diversity (S: 19–35, H'_log2_: 1.2–2.1) [17], and these were similar in range to the fauna associated with mussels (S: 34–46, H'_loge_: 1.5–1.7) along the EPR [19], [37]. Qualitative observations comparing mussel and pompei worm communities at the 9°50′N EPR region revealed two times higher taxonomic richness in mussel beds than in pompei worm aggregations [38]. No quantitative information is yet available for pompei worm associated macrobenthos and for the bare basalt.

The meiofauna (usually defined as the smaller size class of animals and protists passing through a 1 mm sieve and retained on a 63 µm or 32 µm sieve) communities and distribution have been much less studied at vents, although their importance in marine ecosystems has been acknowledged for a long time [39]. The ecological role of meiofauna is often unknown or not considered, and most studies tended to focus on a single habitat and a single higher taxon. Currently, meiofauna species contribute to about 20% of the total diversity known from hydrothermal vents. Meiofauna communities generally exhibit low diversity and species richness, and occur in low population densities [40]. Species diversity of nematodes, one of the prominent meiofauna taxa, was studied in mussel beds growing on basalt along the EPR [41]–[43] and in mussel beds of sedimented vents in the North Fiji Basin [44]. For one of the other important meiofauna taxa, the copepods, qualitative data are available from aggregations of the alvinellid *Paralvinella sulfincola* and the tubeworm *Ridgeia piscesae* colonizing sulfide chimneys at the Juan de Fuca Ridge [45]. A quantitative copepod study compared basalt-hosted mussel and tubeworm aggregations at the EPR [46]. However, thus far only two studies have described the entire meiofaunal communities on a species level from mussel beds at the 11°N EPR and 23°N Mid-Atlantic Ridge and from tubeworm bushes at the 9°50′N EPR [47], [48].

For this study, we identified and quantified the entire meiofauna communities from the main habitat types at the 9°50′N EPR region and documented species diversity, abundance, and distribution according to well-characterized habitat types. Meiofauna data from the tubeworm habitat were already published by the first author in 2007 [47] and are integrated in this study. Also, the nematode and copepod data from the same tubeworm samples were integrated previously in a comparison of nematode and copepod communities separately [43], [46]. Samples covered the entire range of hydrothermal vent fluid regimes from black smoker sulfide chimneys devoid of any visible macrofauna, to pompei worms at black smokers, tubeworms and mussels at basalt, and bare basalt within the AST. By including samples from bare basalt with ambient deep-sea temperature and lack of vent fluid emissions in our study, we can estimate the degree of endemicity of vent meiofauna in this region and discuss underlying ecological and evolutionary processes. By scaling the stress experienced by the animals due to hydrothermal emissions we can test the influence of stress on the meiofauna communities. We studied habitats from extremely high stress levels at bare sulfide chimneys, very high levels at pompei worms, high/intermediate levels at tubeworms, intermediate/low levels at mussels to low levels at bare basalt. Due to different stress regimes we expect distinct meiofauna communities at distinct habitats.

## Methods

### Study area

**Figure 1:**
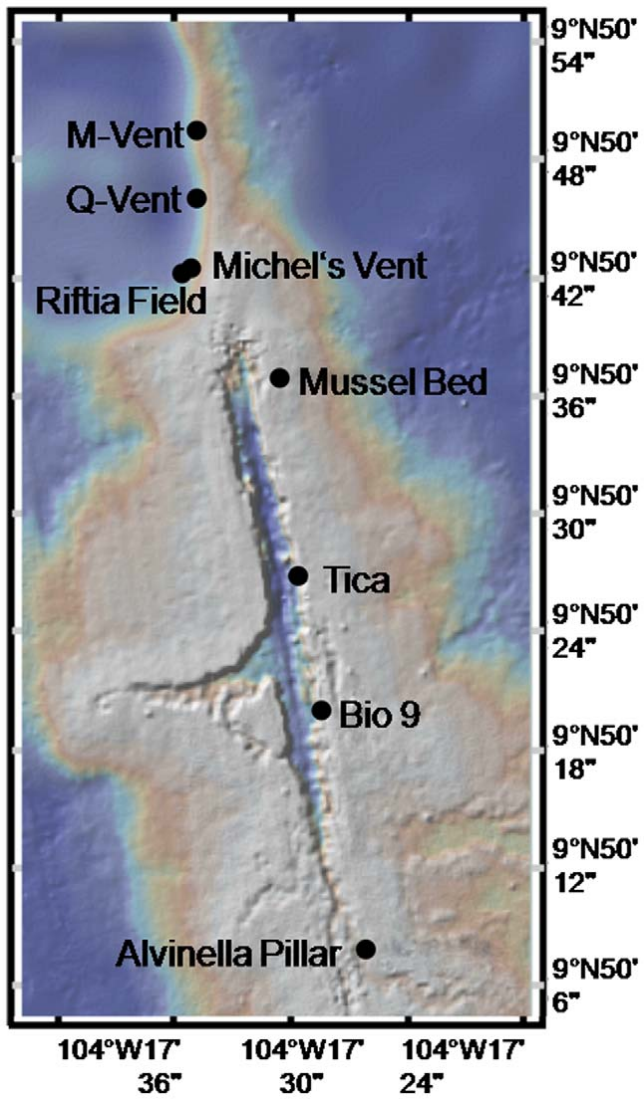
Sample sites within the 9°50′N EPR (East Pacific Rise) region: Alvinella Pillar, Bio 9, Michel's Vent, M-Vent (pompei worm habitats), Tica, Riftia Field (tubeworm habitats), and Mussel Bed (mussel habitat). Bare basalt samples were taken near Tica and Alvinella Pillar.

**Figure 2:**
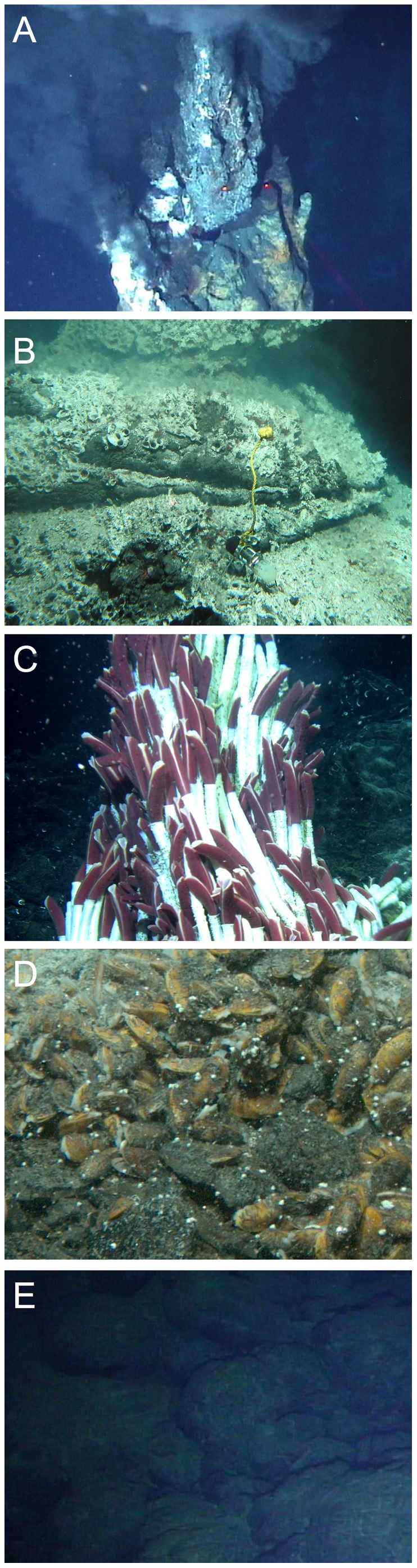
*In situ* photographs of the 5 different habitat types. Sulfide chimney (A), pompei worm habitat with the polychaete *Alvinella pompejana* (B), tubeworm habitat with the vestimentiferan *Riftia pachyptila* (C), the mussel habitat with the bathymodiolin mussel *Bathymodiolus thermophilus* (D), and bare basalt habitat (E).

The study was conducted within the axial summit trough (AST) at the 9°50′N 104°17′W region at the East Pacific Rise (EPR) at 2500 meters depth. A total of 22 samples were taken at 9 sites from 5 different habitat types, using the submersible DSV *Alvin* in the years 2001–2004 (Figure 1, Table 1). All sites were within ∼2 kilometers along the AST. The five different habitat types were chosen accordingly to their different hydrothermal fluid regimes (extremely high, high, vigorous, low, no fluid emissions), termed sulfide chimney (A), pompei worm (B), tubeworm (C), mussel (D), and bare basalt (E) (Figure 2). (A) 4 sulfide chimney samples were collected from active high temperature bare sulfide chimneys of the black smokers P-Vent (2003, AD # 3928, 2510 m, 9°50.287′N, 104°17.487′W), Bio9 (2003, AD # 3929, 9°50.319′N, 104°17.482′W), M-Vent (2003, AD # 3930, 9°50.792′N, 104°17.601′W) and BioVent (2003, AD # 3933, 2505 m, 9°50.927′N, 104°17.584′W). (B) 5 pompei worm samples were obtained at sulfide chimneys of several black smokers colonized by the foundation species *Alvinella pompejana* and *A. caudata* (Michel's-Vent (P1), Alvinella Pillar (P2), Bio 9 (P3), M-Vent (P4, P5)). (C) 6 tubeworms samples were taken at vigorous fluid emissions sites dominated by *Riftia pachyptila* (Tica (T1, T2, T3), Riftia Field (T4, T5, T6)). (D) 3 mussel samples were obtained at a low flow site colonized by *Bathymodiolus thermophilus* (Mussel Bed (M1, M2, M3)), and (E) 4 basalt samples were taken at bare basalt with no hydrothermal fluid emissions, where no foundation species and no visible macrofauna were present (near Tica (B1, B2, B3) and near Alvinella Pillar (B4) in approximate vicinity of 10 m to tubeworms or pompei worms) (Table 1).

**Table 1:** Sample information on geographical location, year of sampling, sample area, and volume of sediment.

Habitat	pompei worm communities					tubeworm communities						mussel communities			bare basalt communities			
Samples	P1	P2	P3	P4	P5	T1	T2	T3	T4	T5	T6	M1	M2	M3	B1	B2	B3	B4
Site	Michel's	Alvinella	Bio9	M-Vent	M-Vent	Tica	Tica	Tica	Riftia	Riftia	Riftia	Mussel	Mussel	Mussel	near	near	near	near
	Vent	Pillar							Field	Field	Field	Bed	Bed	Bed	Tica	Tica	Tica	A.Pillar
Latitide 9°50.	.709′N	.130′N	.335′N	.826′N	.826′N	.447′N	.447′N	.447′N	.705′N	.705′N	.705′N	.615′N	.613′N	.629′N	.447′N	.447′N	.447′N	.130′N
Longitude 104°17.	.585′W	.438′W	.474′W	.583′W	.583′W	.493′W	.493′W	.493′W	.493′W	.493′′W	.493′W	.509′W	.504′W	.512′W	.493′W	.493′W	.493′W	438′W
Depth (m)	2507	2502	2508	2508	2508	2500	2500	2500	2500	2500	2500	2503	2503	2503	2500	2500	2500	2502
Year of Sampling	2004	2004	2004	2004	2004	2001	2001	2002	2001	2001	2002	2002	2002	2002	2003	2003	2004	2004
Alvin dive #	4063	4065	4069	4070	4072	3731	3732	3846	3730	3734	3843	3845	3847	3852	3952	3952	4063	4064
Sample area (cm^2^)	113	343	56	36	17	600	300	700	1300	600	800	1370	770	630	454	263	270	369
Sediment (ml)	17	174	106	138	20	88	165	133	40	37	85	25	15	19	4.9	9.6	2.3	6.5

We named samples after habitat types and their foundation species (P pompei worm, T tubeworm, M mussel) or their substrate (B bare basalt); dominant foundation species at sites were *Alvinella pompejana* Debruyéres & Laubier 1980, *Riftia pachyptila* Jones 1981, and the mytilid mussel *Bathymodiolus thermophilus* Kenk & Wilson 1985. High temperature sulfide chimney habitats are not shown because not a single specimen was detected within these samples and this habitat type was therefore excluded from all analyses.

### Physico-chemical measurements of vent fluid emissions

Prior sampling, temperature was measured *in situ* at all collection sites. At sulfide chimneys, pompei worm, and bare basalt habitats we used the temperature probes of the DSV *Alvin.* At tubeworm and mussel sites, temperature, pH, and sulfide (∑ H_2_S) were measured and data are published in Le Bris et al. [25]. Briefly, temperature and pH were recorded using a glass-Ag/AgCl electrode linked to a thermocouple. Sulfide concentrations were analyzed using the ALCHIMIST (for details see [25]).

### Sample processing

Due to difference in habitat structure it was necessary to use different sampling devices. Tubeworm samples were taken with the hydraulically actuated collection device, named 'Bushmaster Jr. ' lined with a net of 63 µm mesh size [17], [47]. Mussel samples were scooped carefully down to the bottom with a linen bag (63 µm) strengthened at its opening by a steel frame and closed by turning the bag. Sulfide, pompei worm, and bare basalt samples were taken with the hydraulic arm of *Alvin*. A piece of the substrate was very carefully broken off from the habitat and put into the sampling box. Some organisms might have been lost during the approximately 1 meter long transfer to the sampling box on *Alvin*, probably resulting in slight under-sampling of rare species and slight error in species abundances. In all cases the area sampled was photographed before and after sampling in order to estimate the sea-floor sample area of samples taken. Samples were separately put into isolated, previously cleaned plastic boxes on the basket of DSV *Alvin*, transported to the surface, and recovered on deck of the ship R/V *Atlantis*. On board, Bushmaster samples from the tubeworm habitat and mussel scoop samples were sieved through a 1 mm and 63 µm net. Sulfide chimney, pompei worm, and bare basalt samples were sieved additionally through a 32 µm net. In order to check for the presence of fauna in the 32 µm to 63 µm fraction also in the tubeworm habitat, we took in addition to the quantitative Bushmaster collections also qualitative tubeworm samples in the same area and sieved those qualitative samples through a 32 µm and 63 µm net. All samples were fixed in 4% buffered formalin. Samples taken in 2001 and 2002 were transferred to 70% ethanol after one day, but this step was found unnecessary for the quality of fixation and therefore was not done with the samples taken later.

In the lab, all meiofauna animals were sorted, counted, and identified to higher taxa under a dissecting microscope. Sorting revealed that not a single animal was found in the size class from 32 µm to 63 µm in sulfide chimney, pompei worm and tubeworm samples. Only in one out of 4 bare basalt samples a few juvenile nematodes were found, but no new species were detected. These juveniles were excluded from the study to make this sample comparable to all other samples. Thus, we here compare meiofauna in the size range from 63 µm to 1 mm and want to remark that in the size range from 32 to 63 µm usually no fauna was present.

From each sample and each higher taxon (copepods, nematodes, ostracods, acari, foraminiferans) all or at least 300 randomly picked individuals were identified to lowest possible taxon, usually to species level. All species belonging to the permanent meiobenthos (meiofauna living in the benthal and being in the size class of meiofauna as adults according to Giere [39]) were considered in this study. We also recorded the temporary meiobenthos (i.e. species that belong to the macrofaunal size class as adults but are meiofaunal during a certain time of their development i.e. juvenile polychaetes, juvenile gastropods, crustacean larvae). In addition, we detected very few pelagic individuals (Calanoida spp., Corycaeidae spp., Oncaeaidae spp.) in our samples. Both groups (the temporary and pelagic meiofauna) were excluded for further analyses of permanent meiobenthos.

A few individuals of Platyhelminthes and numerous Folliculidae (Ciliophora) were found in some samples but could not be included in further analyses: identification of Platyhelminthes to species level was not possible due to the method of fixation, and distinction between live ciliates and empty tubes was not possible. In the previous study on meiofauna associated with tubeworms we included a single species of Tanaidacea [47], but in the meantime we found that this specific species (*Typhlotanais* sp. 1) can grow to large macrofauna sizes, and we therefore excluded it in this study. Furthermore, the species Harpacticoida sp. 2 in Gollner et al. [47] could be identified as *Xylora bathyalis*.

**Figure 3:**
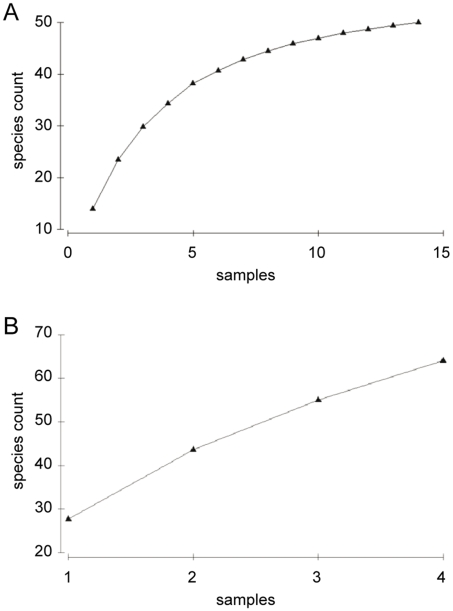
Permuted cumulative species count over samples for vent habitats (A) and bare basalt habitat (B).

For each higher taxon all or at least 300 individuals were identified, for details on slide preparation, literature used for species identification, and species effort-curves see citations in Gollner et al. [47]. Cumulative species-effort curves confirmed that the level of sampling effort per sample and permuted cumulative species counts over samples (number of permutations 999) were sufficient for all studied vent habitats (Figure 3a), but not for the bare basalt habitats (Figure 3b). We are well aware that a total of 4 collected bare basalt samples are insufficient to describe the community on the bare basalt and we expect an increase in species numbers with more sampling in the future. Nevertheless, even this limited bare basalt data set gave us important information for the assessment of endemicity of vent species.

### Quantification of abundance

To compare the variable sample areas of single collections with each other, abundance was standardized to 10 cm^2^ sample area. Standardization to 10 cm^2^ sample area was also used in other hard substrate associated meiofauna studies [49].

Different foundation species (pompei worms, tubeworms, mussels) provided different tubeworm/mussel surface area per sampled area and created different habitat complexity. However, we could not measure the surface area of pompei worms and in consequence standardization to surface area was not possible in this study. Beside the methodological difficulty it is questionable whether or not this standardization is appropriate for meiofauna, since associated macrofauna (i.e. limpets) also offer and increase surface area and living space for the meiofauna, which should then in consequence also be included.

Another standardization for soft substrate meiofauna is done by sediment volume calculations (i.e. core: radius r^2^ x pi x depth). We also measured the volume of accumulated sediment within the foundation species aggregations growing on basalt (see [47]). Standardization of abundance data to 10 ml sediment volume revealed similar abundance values as for 10 cm^2^ sample area standardization at pompei worm and tubeworm habitats and relatively higher abundances at mussel and bare basalt habitats (data not shown). To compare this study with most others we chose to present data based on 10 cm^2^ sample area.

### Data analyses

Species richness (S), Shannon diversity index (H'_loge_), Pielou's evenness index (J'), and expected number of species (ES(100)) were calculated from quantitative species abundance data by DIVERSE subroutines in PRIMER Version 5 package [50]. For statistical analyses bootstrapping (10000 resamplings each, 2-sided t-test, routine “FTBOOT “from the package„ computer intensive statistics”) was used as a well proven method when working with a relatively low number of samples and high variances [51]. We tested for significant differences in abundance (square-root transformed), S (square-root transformed), ES(100) (square-root transformed), H'_loge_ (no transformation), and J' (no transformation). Significance of correlations was carried out by using Pearson's r (F-value and t-value calculations by STATISTICA). All significance levels were classical Bonferroni-corrected (p  =  alpha/n; alpha  = 0.05). To evaluate similarity and dissimilarity of samples, Bray-Curtis similarity was created (abundances of species were standardized and square-root transformed to down-weigh the importance of very abundant species without losing the influence of rarer species). Similarity percentage (SIMPER) analyses, analysis of similarity (ANOSIM), and multi-dimensional scaling (MDS) plot were performed using PRIMER v5. The BIO-ENV procedure was carried out by PRIMER v5 to link biota to multivariate environmental patterns. Maximal temperature, maximal sulfide, and minimal pH were chosen as abiotic variables having possible effect on meiofauna species and communities. Additionally, we included the volume of sediment from each sample as value to test, because it mainly was composed of organic matter, a known food source for meiofauna [47]. We visually analyzed the sediment using a dissection microscope. We found a very high proportion of flocculent organic material (most likely originating from degraded dead animal bodies and bacterial mats), and a very low proportion of inorganic material such as pieces of basalt, minerals from black smokers, or shell remains. Abiotic variables were ln transformed and Euclidean distance was used to create a similarity matrix. For biota, Bray-Curtis similarity from standardized and square-root transformed species abundance data was used. Similarities between biotic and abiotic data were afterwards calculated using Spearman's rank correlation [50].

## Results

### Physico-chemical characteristics

Temperatures were extremely high (244–252°C) at sulfide chimneys lacking macrofauna. At the pompei worm habitat, temperature was highly variable and changing within seconds from overall 14–119°C at the studied sites. In general, in the pompei worm habitat rapid temperature changes can span 40°C [26]. The tubeworm sites Tica and Riftia Field were characterized by warm fluids with maximal temperatures of 32°C and 54°C, respectively. Temperatures were changing within seconds at a scale from 5 to 15°C [25]. At Mussel Bed we measured a maximal temperature of 10°C [25]. On bare basalt the measured temperature was consistently around 2°C, which in this habitat is accompanied by no dectable sulfide and ambient pH of ∼8.1 [31].

We were not able to measure sulfide and pH at the pompei worm habitat prior to sampling. However, several studies from this and other regions of the EPR revealed that the pH is generally acidic with minimal values around pH 4, and sulfide concentrations up to 1520 µM ∑ H_2_S [26], [32], [52]. At the tubeworm and the mussel collection sites, we directly measured these parameters prior to sampling: Tica exhibited maximal sulfide concentrations of 283 µM ∑ H_2_S, and minimal pH of 5.7. No iron was detected in the fluid. At Riftia Field, maximal sulfide concentration was only 95 µM ∑ H_2_S. Minimal pH value was 4.4 in the diffuse flow, and substantial concentrations of dissolved ferrous iron were present at this site (up to 42 µM among the tubeworms). *In situ* analysis of vent fluids at Mussel Bed showed a minimal pH of 6.7 and maximal sulfide of 151 µM ∑ H_2_S [25]. Sulfide and pH values in tubeworm and mussel habitats were changing within seconds and/or minutes [25].

### Abundance

We counted a total of 69 772 individuals from a total sample area >9 000 cm^2^ in 22 samples. The 22 samples were taken from 5 different habitat types (sulfide chimney, pompei worm, tubeworm, mussel, bare basalt) within the AST at the 9°50′N EPR region. Not a single specimen was detected in the sulfide chimney samples. Meiofauna abundance of the other 4 habitats was generally low and varied from 1 to 976 ind. 10 cm^−2^ (Table 2). Abundances were not statistically discernable between pompei worms (mean ± standard deviation: 213±175 ind. 10 cm^−2^) and tubeworms (178±391 ind. 10 cm^−2^), tubeworms and mussels (72±15 ind. 10 cm^−2^), tubeworms and bare basalt (18±23 ind. 10 cm^−2^) habitats. Significantly higher abundances were detected in pompei worms compared to mussels and bare basalt, and mussels to bare basalt habitats. Variations in abundance were higher at sites with higher influence of hydrothermal fluid emissions, and lower at habitats with low or no vent fluids. While abundances of communities at pompei worm and tubeworm habitats ranged from 36–474 and 1–976 ind. 10 cm^−2^, mussel and bare basalt habitats had less abundance variations with 58–87 and 1–51 ind. 10 cm^−2^ (Table 2, 3).

**Table 2:** Meiofauna abundance, relative abundance of taxa, and the diversity measures species richness (S), Shannon diversity (H'_loge_), and Pielou's evenness index (J').

Habitat	pompei worm communities					tubeworm communities						mussel communities			bare basalt communities			
Samples	P1	P2	P3	P4	P5	T1	T2	T3	T4	T5	T6	M1	M2	M3	B1	B2	B3	B4
**Abundance (no. individuals)**																		
Total ab. per sample area	408	7453	1498	252	782	1219	29279	4242	65	60	978	11914	4444	4524	582	141	35	1896
Total ab. 10 cm^−2^	36	217	266	71	474	20	976	61	1	1	12	87	58	72	13	5	1	51
**Relative abundance of taxa (%)**																		
Rel. ab. Nematoda [%]	0	0	0	0	0	78	97	76	31	18	58	49	43	29	4	13	34	1
Rel. ab. Copepoda [%]	99	100	99	99	99	18	2	23	38	80	35	49	53	66	23	63	31	92
Rel. ab. Ostracoda [%]	0	0	0	0	0	0	1	1	0	0	2	1	1	1	1	1	9	1
Rel. ab. Acari [%]	0	0	0	0	0	0	0	0	0	0	0	0	1	0	0	0	0	0
Rel. ab. Foraminifera [%]	1	0	1	1	1	4	1	1	31	2	5	1	2	4	72	23	26	6
**Diversity measures**																		
S total	5	5	4	6	7	11	17	20	10	8	23	29	31	28	32	25	20	34
H'_loge_ total	0.11	0.45	0.18	0.40	0.21	1.13	0.44	1.35	2.00	1.75	1.72	2.34	2.60	2.42	1.18	2.16	2.74	1.42
J' total	0.07	0.28	0.13	0.22	0.11	0.47	0.15	0.45	0.87	0.84	0.55	0.69	0.76	0.73	0.34	0.67	0.91	0.40

P (pompei worm; P1–P5), T (tubeworm; T1–T6), M (mussel; M1–M3), and B (bare basalt; B1–B4).

**Table 3:** Statistical results showing significant differences between habitats.

Habitat	Ab.	S	H'_loge_	J'	ES(100)	Diss. %	R-stat	p
P–T	0.29	**<0.001**	**<0.001**	**<0.001**	**<0.001**	95	1	**0.002**
P–M	**<0.001**	**<0.001**	**<0.001**	**<0.001**	**<0.001**	94	1	**0.018**
T–M	0.81	**<0.001**	**<0.001**	0.09	**<0.001**	68	0.53	**0.024**
P–B	**<0.001**	**<0.001**	**<0.001**	**<0.001**	**<0.001**	93	1	**0.008**
T–B	0.32	**<0.001**	0.20	0.86	0.003	84	0.86	**0.005**
M–B	**0.003**	0.58	0.07	0.24	0.38	75	0.56	0.057

Bootstrapping (bt, 10 000 resamplings each, students t-test) was used to test for significant differences in total abundance 10 cm^−2^ (Ab.), species richness (S), Shannon diversity (H'_loge_), Pielou's evenness (J'), and expected number of species (ES(100)) between the habitats P (pompei worm), T (tubeworm), M (mussel), and B (bare basalt). Significant results after classical Bonferroni-correction are marked in bold. Dissimilarity results (Diss. %) calculated by SIMPER, and ANOSIM results (R-statistics and possible significance level p) are also shown for habitats.

The meiofauna community was composed of Copepoda, Nematoda, Ostracoda, Acari, and Foraminifera (Platyhelminthes and Ciliophora not included in this study). In the majority of our samples, Copepoda was the most abundant higher taxon (1–472 ind. 10 cm^−2^). Within the Copepoda, the Dirivultidae (Siphonostomatoida) and Harpacticoida were the dominant copepod family and order, respectively. Nematodes were absent in the pompei worm habitat. In the other 3 habitats their abundance was highly variable ranging from 1 to 946 ind. 10 cm^−2^. Agglutinated foraminiferans were present at all sites, with a maximum of 9 ind. 10 cm^−2^. Ostracodes were low in abundance (max. 1 ind. 10 cm^−2^) and restricted to tubeworm, mussel, and bare basalt communities. Acari were only found in one mussel sample.

The pompei worm communities were dominated by copepods in relative abundance between 99–100%. A similar, but less pronounced situation was found in the mussel communities (49–66% copepods). No clear pattern was discernible in the tubeworm habitat: in 4 out of 6 samples, nematodes dominated (58–97%), while in 1 sample copepods dominated (80%), and in one other sample nematodes, copepods, and foraminiferans were about equally present. On the bare basalt, copepods dominated the communities in abundance in 2 samples (63% and 92%), while foraminiferans were dominant in one sample (72%), and no taxon dominance was found in another sample (Table 2).

### Diversity

From a total of 22 samples from all studied habitats, 87 species were identified (52 at vent sites, 35 at bare basalt). Looking at higher taxa distribution of species from all samples, 56% of species were copepods, followed by nematodes (30%), foraminiferans (7%), ostracods (6%), and acari (1%). The number of total species found in a habitat increased from pompei worm (11 spp.), to tubeworm (31 spp.), to mussel (36 spp.), and to bare basalt (64 spp.) habitats.

Species richness, expected number of species, and Shannon diversity were in general low and increased from pompei worm (mean ± standard deviation S: 5±1; ES(100): 4±1; H'_loge_: 0.3±0.1), to tubeworm (S: 14±6; ES(100): 11±3; H'_loge_: 1.4±0.6), and to mussel (S: 29±2; ES(100): 19±1; H'_loge_: 2.5±0.1) associated communities and were all statistically significantly different from each other (Table 3). Pielou's evenness was significantly lower at pompei worms (J': 0.1–0.3) compared to tubeworms and mussels (J': 0.2–0.9) (Table 1, 2). Diversity measurements from bare basalt communities (S: 28±6; ES(100): 17±4; H'_loge_: 1.9±0.7, J': 0.3–0.9) were significantly higher than those from pompei worms. Compared to tubeworms, only species richness was significantly higher at bare basalt, and all diversity measurements from mussels were similar to bare basalt (Table 2, 3). The same trend of increasing diversity indices with decreasing influence of hydrothermal fluid emissions was also observed within the nematode and copepod communities (data not shown).

### Community patterns

**Figure 4:**
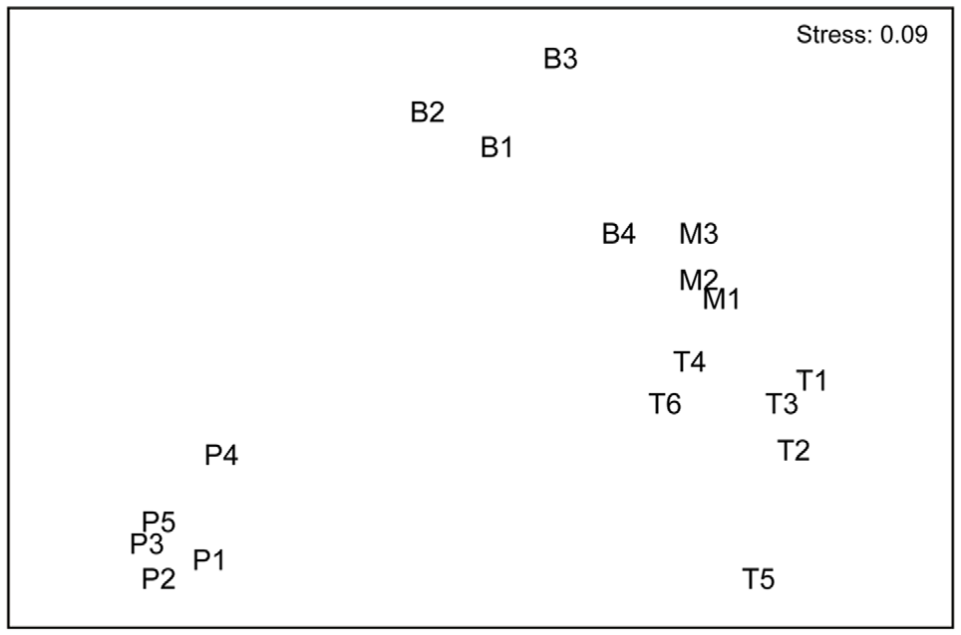
2-dimensional MDS configuration plot for pompei worm (P1–P5), tubeworm (T1–T6), mussel (M1–M3), and bare basalt habitats (B1–B4).

Dissimilarity of pompei worm to tubeworm, to mussel and to bare basalt communities was >93%, (ANOSIM: R = 1; p<0.018). Tubeworm and mussel communities were 68% dissimilar (R = 0.53; p = 0.024), and tubeworm and bare basalt communities showed a dissimilarity of 84% (R = 0.86; p = 0.005). Mussel and bare basalt communities had a dissimilarity of 75% (R = 0.56, p = 0.057; n.s.) (Table 3). Multidimensional scaling (MDS) configuration revealed that meiobenthos from distinct biogenic habitats formed distinct groups (Figure 4).

### Communities and environment

Total meiofauna abundance did not correlate linearly to maximal temperature, maximal sulfide, minimal pH, volume of sediment, and sample area. Maximal temperature was inversely linearly correlated with species richness (r = 0.85; p<0.001) and with Shannon diversity index (r = 0.66; p<0.003). In addition, also minimal pH was linearly correlated with species richness (r = 0.88; p<0.001) and with Shannon diversity index (0.66; p<0.003). An inverse correlation of maximal sulfide with species richness (r = 0.71; p = 0.001) was found, but Shannon diversity (r = 0.59; p = 0.011) was not significantly correlated. Univariate measures of diversity were not correlated with sediment volume and sample area, except for Shannon diversity and sample area (r = 0.69; p = 0.002). BIOENV gave the result that temperature, sulfide, and pH offer the best explanation for community patterns, showing a rank correlation of 0.54. Single abiotic variables which best group the sites, in a manner consistent with the faunal patterns were pH (rank correlation ρ = 0.55), sulfide (ρ = 0.51), temperature (ρ = 0.51), and sediment (ρ = 0.14).

### Meiofauna distribution on a broader scale

We summarized the occurrence of species according to habitat and gave them the following type names: AST generalist (species found on bare basalt and at least in one vent habitat, indicating a broad ecological niche), bare basalt specialist (species only found on bare basalt, indicating a more narrow niche), vent specialist (species only found in one vent habitat), vent generalist (species found in at least two vent habitats, but not on bare basalt). To our study we added available information from other studies to gain a more complete picture [45], [48], [53]–[60] (see Table S1). From the 87 identified species we currently can consider 35 species as bare basalt specialists, 29 species as AST generalists, 12 species as vent generalists, and 11 species as vent specialists. Concerning the species found at vents, 56% of species are AST generalists, 23% are vent generalists, and 21% are vent specialists, so that less than half of species collected for this study from vents can be considered vent endemics.

## Discussion

The AST of the midocean ridge at 9°50′N EPR region houses two fundamentally different ecosystems: (i) the large, continuous basalt which is only scarcely populated by macrofauna with stable, ambient deep-sea temperature and chemistry. (ii) the relatively small, ephemeral, patchy hydrothermal vents at which a distinct zonation of foundation species and their associated faunas along a physical and chemical stress gradient of hydrothermal vent fluids are found. Despite these profound differences, meiobenthos from both ecosystems is more similar than we expected. The majority of species that occur at vents are not endemic, but exist also on the bare basalt. This AST hard substrate, epibenthic and epizooic deep-sea fauna is characterized by a few higher taxa of animals and protists with low diversity and low abundance. Overall, distinct communities colonize each vent habitat in a pattern of diversity inversely correlated with the gradient of hydrothermal vent fluid emissions.

### Low abundance

Abundances below 100 ind. 10 cm^−2^ are common for epizooic vent communities [41], [47], [48], [61]. This feature is shared with epizooic communities from chemosynthesis-based environments such as deep-sea cold seeps and with infaunal communities from sedimented shallow-water vents [62]–[66]. Also, epiphytal communities associated with macroalgae from shallow waters are characterized by low abundances [49]. However, it stands in contrast to infauna from deep-sea seeps and many other non-reducing marine habitats where abundances are on average above 1000 ind. 10 cm^−2^ [39], [67].

At our studied sites, highest epizooic abundance was 976 ind. 10 cm^−2^ in one tubeworm sample, but most samples were characterized by <100 ind. 10 cm^−2^. A similar trend was found in all other comparable epizooic meiofauna studies from deep-sea vents [41], [47], [48], [61]. Also, shallow-water vent meiofauna from sedimented areas exhibits in general low abundances ranging from 0 to a few hundred ind. 10 cm^−2^ [63]–[66]. Interestingly, two of these studies showed that abundances at vents sites were lower compared to control sites [65], [66], while another study revealed the contrary result, i.e. meiofauna abundance increased closer to the vents [64].

Seep epizooic abundance of meiofauna living on tubeworm and on mussel aggregations is similar to those at vents and is also usually <100 ind. 10 cm^−2^ [62]. In contrast, seep infaunal abundance is overall higher (often >1000 ind. 10 cm^−2^), ranging from 1 to 11 000 ind. 10 cm^−2^ (for details see [62], [68]). In seep sediments, it is still unclear whether seeping enhances or diminishes abundance of meiofauna in comparison to the surrounding non-reducing sediments (for details see [69]). Remarkable is the finding that meiofaunal abundance was negatively correlated to macrobenthic abundance in Norwegian seeps [69]. A similar trend was also observed in another seep study, showing meiofauna abundance of ∼1000 ind. 10 cm^−2^ in sediments of pogonophoran fields, but ∼4000 in reduced sediments (one site covered with bacteria). Comparable deep-sea values at the studied region were ∼2300 ind. 10 cm^−2^ [70].

The low meiofauna abundances at deep-sea hydrothermal vents were remarkable, since this productivity rich ecosystem is known to support very high macrofaunal abundances [7]. In addition, meiofauna abundance in the deep sea is in general positively influenced by productivity [67]. Several circumstances might explain the observed low abundances at vent habitats. (1) Bottom-up as well as top-down processes could provide possible explanations for the low meiofauna abundance at hard substrate deep-sea hydrothermal vents. On the one hand, vents are known for their high *in situ* primary production [7], but neither the quality nor the quantity of particulate organic matter (POM), the major food source for meiofauna, has been studied at diffuse flow vents at the 9°50′N EPR. Thus, in theory meiofauna could be limited by food. At the Juan de Fuca Ridge the quality of POM influenced meiofauna distribution [71]. On the other hand, deposit and bacteria feeding meiofauna could be in strong competition for food with the macrofauna. Also highly abundant macrofauna could prey on smaller fauna [72]. (2) Substrate type could be another explanation for low meiofauna abundance of hard substrate deep-sea vents: a shallow-water study showed that meiofauna abundance was lower on rocky shores covered by macroalgae (130–974 ind. 10 cm^−2^), than in sediments (820–6298 ind. 10 cm^−2^) [49]. The authors speculated that the lack of interstitial space, a suitable place for meiofauna to live, could reduce the possibility of colonization on hard substrates. (3) Vent fluid emissions with their high temperatures, low pH and high and toxic sulfide concentrations could also cause low abundances at deep-sea vents. However, in this study highest meiofauna abundance (although of very few, probably well adapted species) was found at sites with high influence of vent fluids. Overall, low meiofauna abundance at deep-sea hydrothermal vents is not understood yet, and various options (vent fluids, bottom-up and top-down processes, influence of substrate type) remain to be tested in the future.

### Low higher taxon diversity

To our knowledge, only 4 metazoan phyla, Arthropoda, Gastrotricha, Nematoda, Platyhelminthes, and 2 protist phyla, Ciliophora and Granuloreticulosa, build the entire permanent meiofauna community in the 9°50′N EPR region. Gastrotricha were described from artificial devices deployed in this area [73], but maybe due to general rarity of this taxon at vents, we did not encounter them in our study of natural communities. The very low higher taxon diversity is striking und not yet understood. Possible explanations could include suitability of substrate type. Many higher taxa are solely reported from sedimented but not from hard substrate ecosystems [39]. In addition, the vent fluid emissions could prevent settlement of higher taxa that are sensitive to high temperatures, low pH, and/or high sulfide concentrations and the variations of these parameters.

The dominance and the high species richness of copepods at hard substrate hydrothermal vents are extraordinary. Usually, nematodes dominate meiofauna communities in abundance and also in species richness [39]. However, it has to be clarified that the large majority of meiofauna studies is performed in sediments, to which nematodes are perfectly adapted with their long and slender bodies. In contrast to those studied sediments, hydrothermal vents at our studied region are found on basaltic hard substrate. Hard substrate communities are often dominated by copepods, since this taxon can climb and crawl overall better than nematodes [39]. Copepoda is the most species rich taxon in our study with 49 identified species. With about 80 described species, it is also one of the most diversified taxa at hydrothermal vents, contributing more than 15% of the animal species documented from vents worldwide [40], [58]. The high species richness of copepods at deep-sea hydrothermal vents is mainly due to the species rich copepod family Dirivultidae which is supposed to be well adapted to the vent environment [46], [74].

### Microbial symbiosis in meiofauna

Hydrothermal vents became famous with the discovery of large, symbiont-housing animals like the giant tubeworm *Riftia pachyptila*, and today many vent species are known to harbor epi- or endosymbiotic bacteria [7], [12], [13]. Symbioses with meiofaunal hosts are rare at basaltic vents, where the lack of sediment does not allow colonization of typical infaunal meiofauna symbioses, such as stilbonematin nematodes or gutless oligochaetes common in suitable coastal shallow-water sands [75]–[77]. Stilbonematins are also found in sedimented shallow-water vents [63], [78], but to our knowledge never at hard substrate deep-sea vents.

Instead, taxa such as folliculids colonizing hard substrates or various living surfaces and especially large solenogastres living on animals are reported from basaltic vents and only there they live in symbiosis with microbes. Recently, a colonial, sessile folliculid ciliate with endo- and ectosymbiotic bacteria was described from Juan de Fuca Ridge [79] and was also present in our samples from EPR. Further, *Helicoradomenia* ssp., an about 2 mm long Solenogastres associated with a consortium of epi- and endocuticular Eubacteria, was reported from tubeworm bushes at Tica at the 9°50′N EPR and other vent habitats [80], but in our study they fell in the macrofauna size class using a 1 mm net [17].

### Diversity

Diversity can be influenced by numerous factors such as disturbance, stress, productivity, and/or habitat modification by foundation species [81]. Meiofauna diversity at deep-sea hydrothermal vents is probably most effected by the environmental stress caused by the exposure to high and variable vent fluid emissions. Diversity measures, such as species richness, were inversely linearly correlated to maximal temperature or sulfide concentrations. A BIOENV analysis showed that 54% of the community pattern variation can be explained by the abiotic factors temperature, pH, and sulfide. These analyses also show that physico-chemical stress is not the only factor shaping the meiofauna community. Disturbances such as frequent volcanic eruptions might be a cause for the overall low diversity of communities in the AST. The influence of productivity on meiofauna diversity is difficult to scale because detailed productivity measurments are lacking. The relatively high diversity in the low heterogeneic bare basalt habitat suggests that habitat heterogeneity might not be the main driver of diversity in the AST.

Physico-chemical characteristics of vent fluid emissions impose several physiological stresses. Our results show that with increasing vent fluid emissions and increasing amplitude of fluctuations, fewer meiofauna species seem to be able to cope with the extreme conditions. Extreme vent fluid regimes with temperatures around 250°C as measured on the surface walls of several black smoker chimneys are above the limits for eukaryotic life, currently thought to be about 45–55°C [82]. These samples lacked any fauna. The foundation species *Alvinella pompejana* and *A. caudata* thrive at the most extreme of vent habitats still populated by animals [26]. Species richness was very low in the pompei worm habitat and mostly copepods, such as the dirivultid *Stygiopontius hispidulus,* were apparently able to live in this unstable habitat. There, animals have to tolerate or have to be able to escape from temporal peaks of high vent fluid emissions. Dirivultid copepods exhibit adaptations to the vent environment such as hemoglobin with a very high and temperature sensitive oxygen affininity [83], [84]. Copepods are also considered as relatively fast compared to other meiofauna taxa. Observations of live vent animals in petri dishes revealed extremely slow moving nematodes but very hectic, fast moving copepods (SG, MB pers. obs.). The agility of copepods might be one of the factors allowing them to invade this habitat. They can escape quickly when conditions change, or can quickly recolonize available free space after i.e. a previous high temperature peak event had killed animals. The more sluggish nematodes, ostracods, and acari are apparently not capable of living in the hot pompei worm habitat. In less extreme and less variable hydrothermal settings such as in tubeworm and mussel habitats, in addition to the relatively fast moving copepods also nematode and ostracod species can establish. Overall, the observed inverse correlation of meiofauna diversity with increasing influence of hydrothermal fluid emissions was also detected within the two most dominant taxa, the Copepoda and the Nematoda.

Previous studies on nematode and copepod communities at deep-sea hydrothermal vents are consistent with our results. The nematode community at sedimented vents in the North Fiji Basin, showed lower species diversity in the center of hydrothermal activity (12–24 spp.) than in nearby areas without vent fluid emissions (55 spp.) [44]. A copepod community study at *Ridgeia pisceae* tubeworm habitats at the Juan de Fuca Ridge also revealed, that sites with low or undetectable vent fluids harbor more copepod taxa than sites with higher fluid emissions [45]. Also a very species-poor copepod community was found at sulfide chimneys colonized by *Paralvinella sulfincola*, comparable to what we found at the chimneys colonized by pompei worms. In *P. sulfincola* aggregations the copepod *Stygiopontius quadrospinosus* instead of *S. hispidulus* dominates the communities with 80% relative abundance [45]. Nematode species richness in mussel beds of other geographical locations is similar to what we detected (12–24 spp., 10 spp., 17 spp. respectively) [41], [43], [44]. In all vent habitats, the most dominant copepod genera belonged to the Dirivultidae (for details see [85]). Among nematodes, the common deep-sea genus *Thalassomonhystera*, dominated in mussel and tubeworm habitats (for details see [68]).

Volcanic eruptions are major disturbances for all species inhabiting the AST. In the here studied region, volcanic eruptions occurred in 1991 and 2006, covered large areas of the AST with lava, and killed the majority of living beings in the area [23], [86]. The influence of volcanic eruptions on meiofauna communities and the meiofaunal successional patterns, the non-seasonal, directional continuous pattern of colonization and extinction [87], are not yet observed. In general, succession is characterized by an increase of species richness and a shift in species composition [88]. Our samples were collected from 2001 to 2004 and were overall species poor. This may suggest that in this studied region, species rich late successional stages might never establish due to the frequent volcanic eruptions.

Deep-sea hydrothermal vents are fueled by high *in situ* primary production via chemosynthesis, and are among the most productive ecosystems in the deep sea [7]. It is well recognized that productivity influences diversity [89], and a frequently cited hypothesis suggests an unimodal diversity pattern along an increasing productivity gradient [90], [91]. Unfortunately, there are no productivity data available for the here studied vent habitats and the bare basalt. Thus, we cannot scale productivity and its possible influence on diversity. It will be an important future challenge to be able to measure productivity at diffuse flow vents.

The physical structure of foundation species alters the enviroment, can facilitate species co-existence and can increase species richness [10]. At hydrothermal vents, prominent foundation species such as pompei worms, tubewoms, or mussels occur in high densities [7]. According to ecological theory, associated macrofauna species richness increased with increasing surface area provided by tubeworms [17]. However, in this study on meiofauna sharing the same samples, this pattern was not observed [47]. In general, little is known on the possible effect of habitat heterogenity on meiofauna species richness. One difficult problem to overcome is to measure surface area enrichment, as it is experienced by the small meiofauna. For such small animals, not only large foundation species but also associated macrofauna alter the environment, and can enhance the potential ecological niche. In our study we were not able to solve this difficulty. However, we found a relatively high meiofauna species richness in the obviously low heterogenic bare basalt, where no foundation species and hardly any macrofauna occured. This suggests that habitat heterogenity might not be the main driver of species richness in the AST.

On a global scale, the habitats in the AST enhance habitat heterogenity and diversity in the deep sea. Total observed nematode genera richness at deep-sea hydrothermal vents is 32, however only 2 of them were restricted to vents [92]. The dirivultid copepods, only found at vents and on bare basalt within the AST, considerably contribute to deep sea diversity with currently 50 described species [85].

The trend that diversity is in general low at deep-sea hydrothermal vents and that diversity is lower at vents than at close-by control sites could also hold true for shallow-water vents and for deep-sea seeps. However, the large majority of these studies was carried out on higher taxon level and only a few detailed studies restricted to nematodes or foraminiferans are available for comparison sofar. For deep-sea whale and wood falls, information completely lacks so far. Nematode species richness was lower in shallow-water vents in the Mediterranean and Pacific (3 and 11 spp.) than control sediments (19 and 22 spp.) [65], [66]. A similar trend was found for Foraminifera communities where forminferans were absent at high temperature vents [93]. Seep sediments in Central Japan contained 28 nematode species, but 44 species were identified from control sediments [94]. Sediments in the center of cold seeps along the Norwegian margin were dominated only by one or two nematode species (total observed genera 19±6), while seeps inhabitated by siboglinid tubeworms and control sites harboured equally genus rich communities with different dominating genera (seep 64±9, control 66±6 nematode genera) [69].

### AST meiofauna and possible underlying ecological and evolutionary processes

More than half of the 52 species found at the 9°50′N EPR vents also inhabit the bare basalt. Taking into account that we only were able to collect 4 samples at the bare basalt, we expect the number of species currently listed as vent endemics (vent specialists and generalists) to decline with more collections in future. However, we can already see a rough outline of underlying different life histories concerning dirivultid and harpacticoid copepods and nematodes. Some species of the Dirivultidae, a family formerly classified as vent endemic [74], must now be considered as AST generalistic, as we also encountered them on bare basalt. Interestingly, many of these Dirivultidae showed relatively higher abundance at vent sites. Many different dirivultid species were found in more than one vent sample while at the bare basalt most species were only detected in a single sample. In contrast, harpacticoids, present in many other marine benthic habitats [39], were usually more abundant and diverse on bare basalt. Nematodes were also more diverse on bare basalt, e.g. no species was found in the pompei worm habitat, and only very few species were present in the tubeworm habitat (e.g. *Halomonhystera hickeyi*, *Thalassomonhystera fisheri*). These few nematode species can become very abundant at vent habitats, suggesting that some species and/or genera have successfully adapted to the vent environment. Whether the success of vent species is due to physiological adaptations, a very broad physiological tolerance, or due to biological interactions remains to be tested.

Disturbances, such as the waxing and waning of vents and even volcanic eruptions, are less dramatic for AST generalists as populations are present nearby on the bare basalt than for species restricted to vents. Vent generalists have the advantage over vent specialists that shifts in vent fluids are tolerated according to the range of physiological capabilities of each species. The number of vents potentially acting as a source for colonization is much higher for generalists, than specific vent types within a given area are for specialists. Consequently, the few vent specialists restricted to one specific habitat are most threatened by disturbances.

Very few bare basalt samples were taken in the vicinity of vents about 10 meters away from vents, and it is far too early to predict which communities are generally found in this neglected habitat. It has to be noticed that the studied bare basalt, although not directly exposed to vent fluids, might exhibit enhanced food sources compared to more distant bare basalt. We identified species that are present on bare basalt but also at vent sites (AST generalists), but there were also true bare basalt specialists. One of those bare basalt specialists is the harpacticoid copepod *Smacigastes barti*, a species found on bare basalt and on artifical tubeworms (pvc hoses mimicking *Riftia pachyptila* tubes) placed on the bare basalt, but never observed at vent sites or on artificial tubeworms positioned within vent sites [53], [95]. It is likely, that the bare basalt specialist meiofauna and/or AST meiofauna extends further into the flanks of the mid-ocean mountain chain until a switch from an epibenthic to an infaunal community occurs due to an increase in sediment coverage.

### No endemic meiofauna in chemosynthetic driven ecosystems?

In contrast to vent macrofauna, where the majority of species is restricted to deep-sea hydrothermal vents [14], the vent meiofauna seems to be a subset of the surrounding AST fauna. In this study the majority of species found at vents, was also present on nearby bare basalt. Nematode genera composition was similar at vent sites and control sites in a sedimented deep-sea hydrothermal vent, but none of the species was common at both sites [44]. No specialized meiofauna, i.e. new genera, families (as for example vestimentiferans in the larger size class) have been detected in hydrothermal vents. Nematode genera occuring at vents and on bare basalt are also known from deep-sea plains, suggesting local adaptation rather than long distance distribution of nematodes (for details see [68]). Harpacticoid copepod genera such as *Ameira*, *Amphiascus*, *Halectinosoma*, or *Halophytophilus* are rarely observed in deep-sea sediments (PMA pers. obs.) but are found associated with deep-sea corals [96], suggesting that probably substrate type could play an important role in harpacticoid copepod distribution. Only the copepod family Dirivultidae might be an exception, being highly successful at vents, present only in low abundance on the nearby bare basalt, and being absent from any other ecosystems (for more information see [85]).

Shallow-water vent meiofauna are also a subset of surrounding sediment fauna [65], [66]. This is in accordance to the macrofauna pattern found in this ecosystem [97]. Also most seep nematode species seem to be related to nematodes from shallow-water environments [68], [69], [94]. Interestingly, a highly dominant nematode species (*Halomonhystera disjuncta* complex) found in bacterial mats at deep-sea seeps has been described from intertidal habitats before. Also the nematode *Terschellingia longicaudata*, a species already known from oxygen poor shallow-water environments, was detected in cold seeps [69]. Harpacticoid copepod genera encountered at deep-sea cold seeps are usually not found or not prominent in sediments of abyssal plains, but are sometimes known from shallow-water habitats (PMA pers. obs.).

### Conclusions

Meiofauna from deep-sea hydrothermal vents occurs in low abundances (∼100 ind. 10 cm^−2^), which is in stark contrast to the macrofauna. Meiofauna abundances from shallow-water vents are also low. Seep epifauna abundance is similar to vent epifauna, but seep infauna abundances are often higher. Deep-sea hydrothermal vent meiofauna diversity is in general low and increases with descreasing influence of vent fluid emissions. Shallow-water vents and cold seeps often showed dimished diversity in comparison to control sites. Interestingly, many meiofauna genera and even some species from hydrothermal vents are well known from other ecosystems, which is contrary to what is known for most macrofauna. These different patterns of meio- and macrofauna abundance, diversity, and distribution patterns at hydrothermal vents are fascinating and not understood yet. It will be a future challenge to unpuzzle those patterns, and to observe evolution of vent fauna from different sizes perspective.

## Supporting Information

Table S1 Distribution of meiobenthic species in the habitats P (pompei worm), T (tubeworm), M (mussel), and B (basalt). The occurrence of species (indicated by x) in their habitats in this study is compared to those of other studies: M* corresponds to the study of Zekely et al. 2006 [48] who studied meiobenthic communities at the mussel site Buckfield at 11°N EPR. Other findings show additional occurrences of species. Reference (ref) is given for each habitat finding. The taxon is given for each species (S  =  Siphonostomatoida: all found species except *Ecbathyrion prolixicauda* belong to the family Dirivultidae), H  =  Harpacticoida, N  =  Nematoda, O  =  Ostracoda, F  =  Foraminifera, A  =  Acari). The type summarizes the overall occurrence of species in their habitats known so far: AST G  =  axial summit through generalist (species found on bare basalt and at least in one habitat at vents), B S  =  basalt specialist (species only found on bare basalt), V S  =  vent specialist (species only found in one habitat at vents), V G  =  vent generalist (species found in at least two habitats at vents and not on bare basalt).
(0.02 MB PDF)
