# Supplementary material for: Diversity of Meiofauna from the 9°50′N East Pacific Rise across a Gradient of Hydrothermal Fluid Emissions
Source: PLoS One. 2010 Aug 10;5(8):e12321. doi: 10.1371/journal.pone.0012321 (PMC2938375; doi:10.1371/journal.pone.0012321)
Supplement: Table S1 — Distribution of meiobenthic species in the habitats P (pompei worm), T (tubeworm), M (mussel), and B (basalt). The occurrence of species (indicated by x) in their habitats in this study is compared to those of other studies: M* corresponds to the study of Zekely et al. 2006 [48] who studied meiobenthic communities at the mussel site Buckfield at 11°N EPR. Other findings show additional occurrences of species. Reference (ref) is given for each habitat finding. The taxon is given for each species (S = Siphonostomatoida: all found species except Ecbathyrion prolixicauda belong to the family Dirivultidae), H = Harpacticoida, N = Nematoda, O = Ostracoda, F = Foraminifera, A = Acari). The type summarizes the overall occurrence of species in their habitats known so far: AST G = axial summit through generalist (species found on bare basalt and at least in one habitat at vents), B S = basalt specialist (species only found on bare basalt), V S = vent specialist (species only found in one habitat at vents), V G = vent generalist (species found in at least two habitats at vents and not on bare basalt). (0.02 MB PDF) [file pone.0012321.s001.pdf]

Table S1. **Distribution of meiobenthic species in the habitats P (pompei worm), T (tubeworm), M (mussel), and B (basalt).**

**Species**	**taxon**	**TYPE**	**P**	**T**	**M**	**B**	**M***	**other findings**	**ref**
Aphotopontius limatulus	S	AST G			x	x	x	site Clam acres (with clams?)	[54]
Aphotopontius mammillatus	S	AST G		x	x	x	x	vestimentiferans	[58]
Aphotopontius probolus	S	AST G		x		x		mussel, vestimentiferans, crab trap	[56]
Ceuthoecetes introversus	S	AST G		x	x	x		vestimentiferans and *Calyptogena*	[58]
Ceuthoecetes acanthothrix	S	AST G		x	x	x	x	vestimentiferans and *Calyptogena*	[58]
Ecbathyrion prolixicauda	S	AST G	x		x	x	x	no	
Scotoecetes introrsus	S	AST G		x	x	x	x	vestimentiferans	[58]
Stygiopontius flexus	S	AST G	x	x		x		siboglinids	[58]
Stygiopontius hispidulus	S	AST G	x	x		x		bivalves, polychaetes	[58]
Stygiopontius paxillifer	S	AST G	x		x	x		site Clam acres (with clams?)	[55]
Stygiopontius quadrispinosus	S	AST G				x		sulfides, vestimentiferans, no flux	[45]
Ameira sp. 1	H	AST G	x		x	x		no	
Amphiascus sp.1	H	AST G			x	x		no	
Halectinosoma sp. 1	H	AST G	x	x	x	x	x	no	
Halophytophilus sp.1	H	AST G	x			x		no	
Xylora bathyalis	H	AST G		x	x	x		no	
Anticoma sp.1	N	AST G			x	x	x	no	
Chromadorita sp.1	N	AST G		x	x	x	x	no	
Halomonhystera hickeyi	N	AST G		x	x	x		no	
Leptolaimus sp.1	N	AST G			x	x	x	no	
Microlaimus sp.1	N	AST G			x	x		no	
Paralinhomeus sp.1	N	AST G			x	x	x	no	
Thalassomonhystera fisheri	N	AST G		x	x	x	x	no	
Prionotoleberis styx	O	AST G				x		site Clam acres (with clams?)	[59]
Thomontocypris gollnerae	O	AST G		x	x	x		no	
Thomontocypris brightae	O	AST G		x	x	x		no	
Xylocythere vanharteni	O	AST G		x	x	x		no	
Abyssotherma pacifica	F	AST G	x	x	x	x		no	
Deuterammina sp. 1	F	AST G		x	x	x		no	
Exrima sp. 1	S	B S				x		no	
Ameiropsis sp.1	H	B S				x		no	
Argestes sp.1	H	B S				x		no	
Atergopedia sp.1	H	B S				x		no	
Ectinosoma sp. 1	H	B S				x		no	
Ectinosoma sp. 2	H	B S				x		no	
Idomene sp. 1	H	B S				x		no	
Idomene sp. 2	H	B S				x		no	
Idomene sp. 3	H	B S				x		no	
Leptotachidia sp. 1	H	B S				x		no	
Mesochra sp. 1	H	B S				x		no	
Sarsameira sp. 1	H	B S				x		no	
Smacigastes barti	H	B S				x		on artificial substrates on basalt	[53]
Stenocopia sp. 1	H	B S				x		no	
Stenocopia sp. 2	H	B S				x		no	
Stenocopia sp. 3	H	B S				x		no	
Astomonema sp. 1	N	B S				x		no	
Chromadora sp. 1	N	B S				x		no	
Chromadorida sp. 1	N	B S				x		no	
Chromadorida sp. 2	N	B S				x		no	
Euchromadora sp. 1	N	B S				x		no	
Eurystomina sp. 1	N	B S				x		no	
Metoncholaimus sp. 1	N	B S				x		no	
Microlaimus sp. 2	N	B S				x		no	
Monhysterida sp. 1	N	B S				x		no	
Monhysterida sp. 2	N	B S				x		no	
Paracyatholaimus sp. 1	N	B S				x		no	
Paracyatholaimus sp. 2	N	B S				x		no	
Prooncholaimus sp. 1	N	B S				x		no	
Rhabdocoma sp. 1	N	B S				x		no	
Syringolaimus sp. 1	N	B S				x		no	
Tripyloides sp. 1	N	B S				x		no	

Species	taxon	TYPE	P	T	M	B	M*	other findings	ref
Foraminifera sp. 2	F	B S				x		no	
Foraminifera sp. 3	F	B S				x		no	
Foraminifera sp. 4	F	B S				x		no	
Aphotopontius acanthinus	S	V G		x				on crabs, limpets, bac. mats	[57]
Aphotopontius arcuatus	S	V G	x		x		x	bivalves	[58]
Aphotopontius flexispina	S	V G		x				bivalves	[58]
Ceuthoecetes aliger	S	V G		x	x		x	vestimentiferans and *Calyptogena*	[58]
Nilva torifera	S	V G			x		x	vestimentiferans and *Calyptogena*	[58]
Rhogobius contractus	S	V G		x	x		x	no	
Stygiopontius sentifer	S	V G	x				x	polychaetes, in tubes of *Alvinella*	[58]
Stygiopontius stabilitus	S	V G	x	x				*Paralvinella*	[56]
Bathylaophonte pacifica	H	V G		x			x	invertebrates	[58]
Daptonema sp. 1	N	V G		x	x			no	
Halomonhystera sp. 1	N	V G		x	x		x	no	
Polycopetta pax	O	V G		x	x			no	
Aphotopontius hydronauticus	S	V S		x				no	
Benthoxynus tumidiseta	S	V S		x				vestmentiferans	[60]
Stygiopontius mucroniferus	S	V S		x				no	
Ameira sp. 2	H	V S			x			no	
Bradya sp. 1	H	V S			x			no	
Diosaccinae sp.1	H	V S			x			no	
Harpacticoida sp. 1	H	V S		x				no	
Tetragoniceps sp. 1	H	V S			x			no	
Paracanthonchus sp.1	N	V S			x		x	no	
Forminifera sp. 1	F	V S		x				no	
Lohmannella sp. 1	A	V S			x			no	
